# Scientific Items

**Published:** 1879-12-20

**Authors:** 


### SCIENTIFIC ITEMS.

**A Curious Telephonic Phenomenon.**—Dr. C. E. Chase, of Montezuma, Cayuga county, New York, sends us the following note:

I wish to call attention to a phenomenon in regard to transmission of sound by telephone that I have never yet seen brought to notice. In January, 1878, myself, another gentleman, and the operator were in the office of the Western Union, at this place, between nine and ten in the evening, when our attention was drawn by hearing singing, which seemed, although at a distance, to be sweet and distinct. Stepping out of the office to listen to the music, we found it to cease suddenly, but on returning into the office it was resumed. On investigation we found the sounds proceeded from the *relay* connected with the wire. We distinctly heard "Sweet Home," several familiar operatic airs, "Put me in my little bed,," etc. We found, on looking the matter up, that our singer was in Syracuse, a distance of some thirty miles away, and that Syracuse and Rochester were united by telephone.

The point is, we caught the sounds, without a telephone, at a distance of some thirty miles, with no other instruments except such as are ordinarily used in the offices of the Western Union. This relay is one of their new ones of 156 ohms. It stands upon a desk about five feet long, two and a half feet wide, and eight inches deep, used for keeping papers, etc. This would help to prolong and intensify the sound. I fully understand the construction of the ordinary telephone, and can readily understand its action through acoustic principles, but here is a phenomenon, which, if understood, is not so easily explained. I do not know the kind of telephone used in this case, but could probably ascertain, as I have the name of the operator at the Syracuse end of the line. If you can explain this matter, or if any of our readers know of a like occurrence, I should be glad to hear from them.—*Journal of Chemistry.*

**Indestructibility of Matter.**—A glass tube two centimetres wide and fifteen long is closed at one end like a test tube, but drawn out at the other. In it is put two centigrams of freshly ignited charcoal. It is now filled with dry oxygen, and sealed and weighed. By heating it gently on the rounded end the charcoal takes fire, and may, with care, be entirely burned without breaking the tube. When the charcoal has wholly vanished it is weighed again, and of course, no change of weight is noticed.—*Ibid.*

**Diffusion of Gases.**—To show how slowly one gas diffuses into another gas, Von Than takes a tall and narrow glass cylinder, and attaches to the bottom inside a long strip of white filter paper which has been saturated with lead acetate. The clyinder is inverted over a hollow stopper, or other vessel containing a few drops of sulphydric acid solution. The lower end of the paper is not blackened for ten minutes or more, and the blackening proceeds but slowly towards the other end. Iodide of starch paper and chlorine water may also be employed.—*Ibid.*

**Diffusion of Gases through Collodion Membrane.**—A piece of collodion balloon is fastened over the top of a funnel, which is then inverted and connected by a bent glass tube with a V-tube, into the bend of which is melted a piece of platinum wire. A little mercury is poured into the V-tube, and a second wire is arranged so as almost to touch the surface of the mercury. On connecting these wires with two poles of a battery, no current will pass. The inverted funnel is now brought into a vessel of carbonic acid, when this gas slowly diffuses into the funnel through the collodion membrane. In five or ten minutes the pressure on the collodion will have raised it sufficiently to close the circuit, which can be made to ring an electric bell, or announce itself by heating a fine platinum wire.—*Ibid.*

**Valuable if True.**—The following clipping is going the rounds of the press, and if the statements made are true, it contains information which will be appreciated by the female sex at least:

There are many of our brilliant flowers—such as dahlias, pansies, pinks, geraniums, sweet Williams, carnations, gladiolus—which may be preserved so as to retain their color for years. White flowers will not answer for this purpose, nor any succulent plant, as hyacinth or cactus. Take deep dishes, or those of sufficient depth to allow the flowers to be coverd an inch deep with sand. Get the common white sand, such as is used for scouring purposes, cover the bottom of the dish with a layer half an inch deep, then lay in the flowers, their stems downwards, holding them firmly in place while you sprinkle more sand over them, until all places between the petals are filled, and the flowers buried out of sight. A broad dish will accommodate quite a number. Allow sufficient sand between them. Set the dish in a dry, warm place, where they will dry gradually; and at the end of the week pour off the sand and examine them. If there is any moisture in the sand, it must be dried out again before using; or fresh sand may be poured over again, the same as before. Some flowers will require weeks to dry, while others will become sufficiently dry to put away in a week or ten days. By this simple process flowers, ferns, etc., are preserved in their proper color, which is far better than to press them in books. When arranged in groups or mounted on cards or in little straw baskets, they may be placed in frames under glass.—*Ex.*

**Division of Labor among Ants.**—One of the chief peculiarities of the ants is their social relations. Assembling in countless multitudes, they are divided into different classes, each with a special order of duties to fulfill, but all working harmoniously for a definite end—the perpetuation of the species. Their communities consist of males, females, and neuters; with generally two and sometimes three distinct orders or castes of the latter. Upon them devolves all the labor, the divisions being known as the worker-minors and the worker-majors, the brunt of the work falling upon the first, while the function of the worker-major, though not definitely understood, seems to be that of a superintendent or a soldier, or perhaps a combinatton of the two.—E. R. Keland, in *Popular Science Monthly for July.*

